# Bax inhibitor-1 deficiency leads to obesity by increasing Ca^2+^-dependent insulin secretion

**DOI:** 10.1007/s00109-020-01914-x

**Published:** 2020-05-11

**Authors:** Koenraad Philippaert, Michael Roden, Dmitrij Lisak, Diones Bueno, Tomas Jelenik, Konstantin Radyushkin, Teresa Schacht, Margot Mesuere, Verena Wüllner, Ann-Kathrin Herrmann, Jan Baumgart, Rudi Vennekens, Axel Methner

**Affiliations:** 1 Laboratory of Ion Channel Research, VIB Center for Brain & Disease Research, Leuven, Belgium; 2 Department of Cellular and Molecular Medicine, KU Leuven, Leuven, Belgium; 3 Division of Endocrinology and Diabetology, Medical Faculty, Heinrich Heine University, Düsseldorf, Germany; 4 Institute for Clinical Diabetology, German Diabetes Center, Leibniz Center for Diabetes Research, Heinrich Heine University, Düsseldorf, Germany; 5 German Center for Diabetes Research (DZD), München-Neuherberg, Germany; 6 Institute for Molecular Medicine of the University Medical Center, Johannes Gutenberg University, Mainz, Germany; 7 Mouse Behavioral Unit, The Johannes Gutenberg University, Mainz, Germany; 8 Translational Animal Research Center, The Johannes Gutenberg University, Mainz, Germany

**Keywords:** Bax inhibitor-1, TMBIM6, Obesity, Insulin secretion, Hepatic Steatosis

## Abstract

**Abstract:**

Transmembrane BAX inhibitor motif containing 6 (TMBIM6), also known as Bax inhibitor-1, is an evolutionarily conserved protein involved in endoplasmic reticulum (ER) function. TMBIM6 is an ER Ca^2+^ leak channel and its deficiency enhances susceptibility to ER stress due to inhibition of the ER stress sensor IRE1α. It was previously shown that TMBIM6 overexpression improves glucose metabolism and that TMBIM6 knockout mice develop obesity. We here examined the metabolic alterations underlying the obese phenotype and subjected TMBIM6 knockout mice to indirect calorimetry and euglycemic-hyperinsulinemic tests with stable isotope dilution to gauge tissue-specific insulin sensitivity. This demonstrated no changes in heat production, food intake, activity or hepatic and peripheral insulin sensitivity. TMBIM6 knockout mice, however, featured a higher glucose-stimulated insulin secretion in vivo as assessed by the hyperglycemic clamp test and hepatic steatosis. This coincided with profound changes in glucose-mediated Ca^2+^ regulation in isolated pancreatic β cells and increased levels of IRE1α levels but no differences in downstream effects of IRE1α like increased *Xbp1* mRNA splicing or Ire1-dependent decay of insulin mRNA in the pancreas. We therefore conclude that lack of TMBIM6 does not affect insulin sensitivity but leads to hyperinsulinemia, which serves to explain the weight gain. TMBIM6-mediated metabolic alterations are mainly caused by its role as a Ca^2+^ release channel in the ER.

**Key messages:**

- TMBIM6^−/−^ leads to obesity and hepatic steatosis.
- Food intake and energy expenditure are not changed in TMBIM6^−/−^ mice.
- No changes in insulin resistance in TMBIM6^−/−^ mice.
- Increased insulin secretion caused by altered calcium dynamics in β cells.

## Introduction

The endoplasmic reticulum (ER) is key to the synthesis, folding and sorting of proteins destined for the secretory pathway. ER dysfunction triggers the unfolded protein response (UPR) via the stress sensors protein kinase RNA-like ER kinase (PERK), inositol-requiring protein 1α (IRE1α), and activating transcription factor 6 (ATF6) (reviewed by [1]). The serine/threonine protein kinase and endoribonuclease, IRE1α, is the most ancient and evolutionary-conserved branch of the UPR [2]. Its activation enhances splicing of the mRNA for the transcription factor X-box-binding protein-1 (*sXbp1*) and its translocation to the nucleus where it induces the expression of UPR target proteins but can also induce the degradation of specific mRNAs through a pathway called Ire1-dependent decay (RIDD) [3, 4].

The ER also stores and controls intracellular calcium (Ca^2+^) for a vast array of cellular functions ranging from short-term responses such as contraction and secretion to long-term regulation of cell growth and proliferation [5]. Another evolutionarily conserved protein, transmembrane BAX inhibitor motif containing 6 (TMBIM6), also known as Bax inhibitor-1 (BI-1), bridges Ca^2+^ homeostasis and UPR function of the ER [6]. TMBIM6 is a very hydrophobic protein exerting its anti-apoptotic function mainly in paradigms of ER stress but not against stimulators of mitochondrial or TNF/Fas-death receptor apoptosis pathways [7]. It is the founding member of a family of six evolutionarily conserved proteins located in diverse intracellular membranes [8]. TMBIM6-deficient (TMBIM6^−/−^) mice have continuously increased sXBP-1 levels under conditions of ER stress [9] suggesting an inhibition of IRE1α, maybe via direct interaction of TMBIM6 with the C-terminal region of IRE1α [10]. Moreover, overexpression of TMBIM6 decreases the Ca^2+^ content of the ER [11]. This highly conserved C-terminus of TMBIM6 [8] forms a Ca^2+^ pore, which is sufficient to induce Ca^2+^ leakage from the ER in vitro [12] mediated via a di-aspartyl pH sensor [13, 14], a fact recently corroborated by structural data obtained with a prokaryotic BI-1 ortholog [15].

We have recently shown that the Ca^2+^-modulating activity of TMBIM6 also plays a role in vivo. TMBIM6^−/−^ mice exhibit higher nuclear translocation of nuclear factor-κ light-chain enhancer of activated B cell (NF-κB) proteins along with leukopenia, erythrocytosis, and increased splenic marginal zone B cells, which associate with increased cytosolic and ER-located Ca^2+^, but not with ER stress [16]. Of note, these mice also had increased body weight suggesting a role in metabolism [16]. Likewise, both genetically (ob/ob and db/db) and diet-induced obese mice feature down-regulated TMBIM6 in liver and skeletal muscle [17]. On the other hand, overexpression of TMBIM6 by adenoviral gene transfer dramatically improved glucose metabolism in lean and diet-induced obese mice and reversed hyperglycemia in db/db mice, which was accounted for by lower gluconeogenesis and improved insulin sensitivity.

To better understand the mechanism by which absence of TMBIM6 leads to obesity, we performed a comprehensive metabolic phenotyping of TMBIM6^−/−^ mice comprising indirect calorimetry, euglycemic-hyperinsulinemic, and hyperglycemic clamp experiments. As these experiments suggested hyperinsulinemia-caused obesity, we analyzed glucose-induced Ca^2+^ dynamics in isolated pancreatic islets which demonstrated increased Ca^2+^ levels in young mice as the underlying reason for hyperinsulinemia.

## Materials and methods

### Animals

BI-1 KO mice first described by Chae et al. [7] were identified by genotyping according to the published protocol and backcrossed to wild-type C57/Bl6 mice for more than 15 generations. All mice were maintained in the local animal facility. All animal experiments were fully approved by local authorities for animal experimentation. Mice were age and sex matched and 8–30 weeks old.

### Metabolic cages

Male mice of 8 to 16 weeks old were acclimated in metabolic chambers (TSE Systems, Germany) for 4 days before the start of the recordings. Mice were continuously recorded for 3 days with the following measurements taken every 30 min: water intake, food intake, ambulatory activity (in *X*- and *Z*-axes), and gas exchange (O_2_ and CO_2_) (using the TSE LabMaster system, Germany). vO_2_, vCO_2_, and energy expenditure (EE) were calculated according to the manufacturer’s guidelines (PhenoMaster Software, TSE Systems). The respiratory exchange rate (RER) was estimated by calculating the ratio of vCO_2_/vO_2_. EE was analyzed after normalization by body weight. In addition, food intake was determined continuously by integration of weighing sensors fixed at the top of the cage, from which the food containers have been suspended into the sealed cage environment.

### Glucose clamp tests

All mice received a silicon catheter, which was placed surgically into the right jugular vein under Isofluran (CP Pharma, Burgdorf, Germany) anesthesia and recovered from surgery for 3–5 days. The mice were fasted for 6 h before the clamp experiments. To examine insulin-stimulated glucose metabolism, conscious mice underwent hyperinsulinemic-euglycemic clamps as described before [18, 19]. Briefly, D-[6,6-^2^H_2_]glucose (Cambridge Isotope Laboratories, Andover, MA) was infused for 120 min; then, the clamp test was performed with a primed continuous infusion of insulin (Huminsulin, Lilly, Giessen, Germany) along with D-[6,6-^2^H_2_]glucose for 180 min. Euglycemia was maintained by periodical adjustment of the glucose infusion rates (GIR_NG_) using 20% glucose, also containing D-[6,6-^2^H_2_]glucose to maintain isotopic steady state. Blood samples were taken at 10-min intervals to measure glucose and at 0, 15, 30, 60, 90, and 120 min for measuring insulin levels. Rates of endogenous glucose production (EGP) and whole-body glucose disposal (Rd) during the clamps were calculated as previously described [18, 19].

To examine glucose-stimulated insulin secretion, conscious mice underwent hyperglycemic clamps following a 6-h fast [20]. Target hyperglycemia of 250 mg/dL, approximately doubling the fasting blood glucose, was achieved by periodical adjustment of the infusion rates (GIR_HG_) of 20% glucose solution in 5-min intervals. The blood was sampled at 0, 15, 30, 60, 90, and 120 min for measuring plasma insulin concentrations.

### Isolation of pancreatic islets

The Krebs solution for preparation contained (in mM) 119 NaCl, 4.75 KCl, 1.2 MgSO_4_, 1.18 KH_2_PO_4_, 2.54 CaCl_2_, 5 NaHCO_3_, 20 HEPES, and 5 glucose (pH 7.4). Collagenase P (2 mg/mL) was injected into the pancreatic duct of the mice, followed by dissection of the pancreas. Digestion of the whole pancreas was obtained by shaking for 8 min at 37 °C. After gravitational sedimentation, islets were isolated by three rounds of handpicking. Islets were cultured in advanced RPMI 1640 (Gibco) supplemented with 10% FCS, 100 U/mL penicillin/streptomycin, and 4 mM glutamax at 37 °C and 5% CO_2_, for 16–48 h prior to experimentation.

### Ca^2+^ imaging of pancreatic islets

Pancreatic islets were incubated with 1 μM FURA2-AM for 1 h and mounted on a fluorescence microscope equipped with a heated objective and temperature-controlled multichannel perfusion system, kept at 37 °C. The islets were perfused with a solution containing (mM) 120 NaCl, 4.8 KCl, 2.5 CaCl_2_, and 10 HEPES supplemented with 3 mM of glucose during the first 5 min of the measurement and 10 mM glucose from 5′ to 50′. A total of 30 mM KCl mediated depolarization of the islets and was used as positive control from 50′ to 55′.

### Data analysis

Ca^2+^ oscillation frequency during the application of 10 mM glucose was analyzed. The Fourier transformed frequency (FT) spectrum of the first-time derivative of the Ca^2+^ oscillations was used in further analysis. The relevant oscillation frequencies between 0 and 0.05 Hz were determined. The highest peak represents the dominant oscillation frequency and all frequencies peaking at ≥ 60% of the maximum were considered relevant frequencies. Based on the oscillation frequency, the islets were divided in fast (relevant frequencies only > 0.015 Hz), slow (relevant frequencies only < 0.015 Hz), or mixed (relevant frequencies in both parts of the FT spectrum) oscillating islets. The baseline (F0) was defined as the average fluorescence ratio observed between 100 and 500 s in the 3 mM condition. The AUC was determined by integrating the baseline-corrected fluorescence trace (F-F0). Peak width was determined for all peaks in the oscillation pattern between 20 and 50 min. We determined the peak width as the time between a positive and a negative crossing of the 0.3 level of the normalized residuals of the linear fit. For each islet, we took the average width of all observed oscillations. Islet size was determined with the image-processing software ImageJ. Data was analyzed using MS Excel 2010, IGOR Pro 6.3.7.2, and Origin 8.6. We used the two-tailed two sample *t* test and where necessary corrected for unequal variance between different groups.

### Immunohistochemistry

Animals were perfused and tissues fixed with 4% paraformaldehyde (Carl Roth). ImageJ was used for image analysis: random regions or 0.05 mm^2^ were sampled and the fat content present was determined based on negative hematoxylin eosin staining. Comparison of the different conditions was carried out with a two-way ANOVA and post hoc Bonferroni pairwise comparison.

### Transmission electron microscopy

Cells were pelleted, fixed in 2.5% glutaraldehyde, 2% PFA, and 0.05% tannic acid and afterwards treated with 2% osmium tetroxide. After staining with 1.5% uranylacetate and 1.5% phosphotungstic acid, pellets were embedded in epoxide resin (Spurr) and dissected in 70–80-nm-thick slices on an ultramicrotome (Reichert Ultracut; Vienna, Austria). Images (30 KO and 32 WT) were taken on a Hitachi H 600 transmission-electron microscope.

### Immunoblotting

Pancreata were lysed in ice-cold CelLytic M buffer (Sigma-Aldrich) containing mini complete protease inhibitor cocktail (Roche) and centrifuged for 30 min at 16,000*g*. Fifty-microgram protein lysates were blotted and incubated with primary antibodies (anti-BIP Cell Signaling #3177, anti-IRE1α Cell Signaling #3294, anti-α/β-Tubulin Cell Signaling #2148, anti-sec62 was a kind gift of Richard Zimmermann Saarland University) diluted 1:1000. Secondary antibodies were anti-mouse or anti-rabbit IgG (Fc) infrared fluorescence conjugated secondary antibodies (Licor, 1:30,000). The membranes were scanned for infrared fluorescence at 680 and 800 nm using the Odyssey system (Licor), and the signal was analyzed with the image-processing software ImageJ (NIH).

### RNA preparation and quantitative RT-PCR

Animals were killed by cervical dislocation. All preparation steps were carried out in liquid N_2_-cooled materials. The dissected pancreas were mechanically disrupted with mortar and pestle precooled in liquid nitrogen. Disrupted tissue was further homogenized using the QIAShredder columns (Qiagen). Total RNA was extracted with RNeasy mini kit (Qiagen) according to manufacturer’s instructions. All samples were lysed with RNase-free DNase Set (Qiagen) to eliminate residual DNA. RNA was reverse transcribed using the cDNA synthesis kit (Life Technologies). For amplification and detection of spliced and unspliced XBP1, the Takyon No ROX Probe MasterMix blue dTTP (Eurogentec) was used. Total *Xbp1* (spliced and unspliced) levels were quantitated using probe 60, whereas unspliced *Xbp1* was specifically measured using probe 49 (Universal probe library, Roche) because the reverse primer anneals to the 26 bp that are spliced out. Mouse embryonic fibroblasts treated with 0.5 μM thapsigargin for 24 h served as positive control for the assay. The expression was quantified to the relative levels of the housekeeping gene hypoxanthine-guanine phosphoribosyltransferase (*Hprt*). *Ins1*, *PC1*, and *PC2* mRNA levels were quantified using the FastStart Universal SYBR Green Master (Rox) kit (Roche) using glyceraldehyde-3-phosphate dehydrogenase (*Gapdh*) as housekeeping gene. RNAse free water was used as no template control (NTC). All reactions were performed as triplicate with the CFX Connect Real Time-PCR detection System (Bio-Rad). Analysis of the results was performed using the ΔΔCT method. Primers 60-fw tctgacactgttgcctcttca, 60-rv ctctgggagttcctccagact, 49-fw ctgacgaggttccagaggtg, 49-rev gcagaggtgcacatagtctgag, hprt probe cctctcgaagtgttggatacaggcca, hprt-fw: tcctcctcagaccgctttt, hprt-rev: cctggttcatcatcgctaatc. Ins1-fw gcaagcaggtcattgtttcaac, Ins1-rv aagcctgggtgggtttgg, PC1-fw ctttcgccttcttttgcgttt, PC1-rv tccgccgcccattcatta ac, PC2-fw agagagaccccaggataaagatg, PC2-rv cttgcccagtgttgaacaggt. Mm_Gapdh_3_SG QuantiTect Primer Assay (Qiagen) was used for *Gapdh* mRNA level determination.

### Statistical analysis

Data were analyzed by Graphpad Prism and are shown as mean ± SD. The ROUT test was used to identify outliers, the Shapiro-Wilk test to check for normality. Parametrically distributed data were further analyzed using the Student’s *t* test and nonparametrically distributed data using the Mann-Whitney test. Statistically significant differences were assumed with a *p* < 0.05.

## Results

### Adult TMBIM6^−/−^ mice are obese and develop hepatic steatosis

**Fig. 1:**
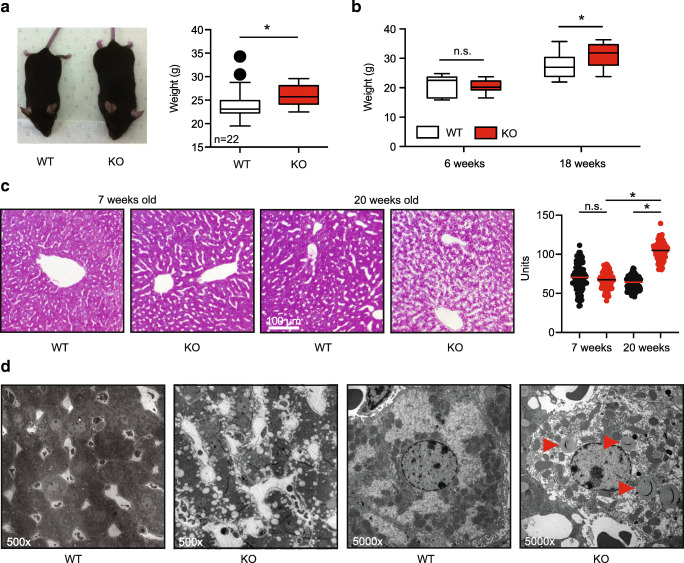
Adult TMBIM6^−/−^ mice are obese and develop hepatic steatosis. **a**, **b** Adult but not young TMBIM6^−/−^ mice are significantly heavier than wild-type (WT) littermates. **a**, **b** Different cohorts of mice. Obesity correlates with the development of hepatic steatosis shown in **c** by representative hematoxylin and eosin staining on liver slices of WT and TMBIM6^−/−^ mice: note the increased presence of unstained regions indicating more fat content in the liver slice of the older TMBIM6^−/−^ mice compared to WT mice and the younger groups. Statistical analysis based on the fat content in randomly sampled 0.05-mm^2^ regions from stained slices in the different conditions (two-way ANOVA with post hoc Bonferroni pairwise comparison). **d** Transmission electron microscopy. Arrows indicate lipid droplets. Normal distribution was assessed using the Shapiro-Wilk test and significant differences calculated using the Student’s *t* test, *p < 0.05

We previously reported that adult TMBIM6^−/−^ (KO) mice kept on normal diet are significantly more obese than their wild-type littermates (Fig. 1a) but did not study this in more detail because we were focusing on changes observed in the immune system [16]. This increased obesity was consistent in a second cohort of mice which we followed over time (Fig. 1b). We noted that the increased body weight of TMBIM6^−/−^ mice develops with adulthood with a significantly higher body weight for TMBIM6^−/−^ at 18 weeks (Fig. 1b). In parallel, liver fat content was increased in TMBIM6^−/−^ at 20 weeks as shown by both conventional histology (Fig. 1c) and transmission electron microscopy (Fig. 1d), suggesting that the increased weight is not caused by an increased muscle mass or size.

### TMBIM6^−/−^ mice do not exhibit altered food intake, activity, or heat production

**Fig. 2:**
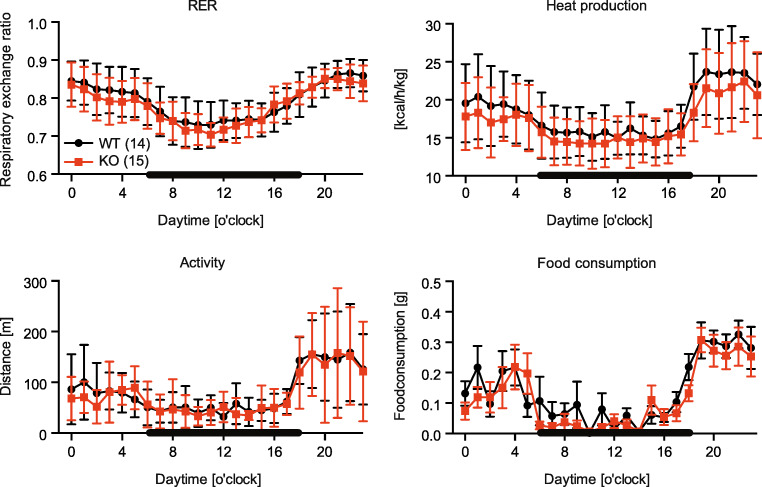
TMBIM6^−/−^ mice do not exhibit altered food intake, activity, or heat production. Respiratory exchange ratio (RER), heat production, activity, and food consumption of the indicated number of WT and KO mice were quantitated by indirect calorimetry in dedicated metabolic cages. Dark and light cycles are indicated

Increased body weight is caused by an imbalance between food intake and energy consumption. We quantitated these factors with a cohort of 14–15 male mice per genotype using dedicated metabolic cages that allow the quantification of the respiratory exchange ratio (RER), heat production, activity, and food consumption. The RER is the ratio between the amount of CO_2_ produced and oxygen consumed and is determined by comparing exhaled gases to room air. In steady state, the RER correlates with the respiratory quotient, an indicator of which fuel (carbohydrate or fat) is being metabolized to supply the body with energy. There were no significant differences in all of these measures between KO and WT animals (Fig. 2) meaning that the differences in body weight are not caused by behavioral differences or a different metabolic use of food.

### No alteration of insulin sensitivity in TMBIM6^−/−^ mice

**Fig. 3:**
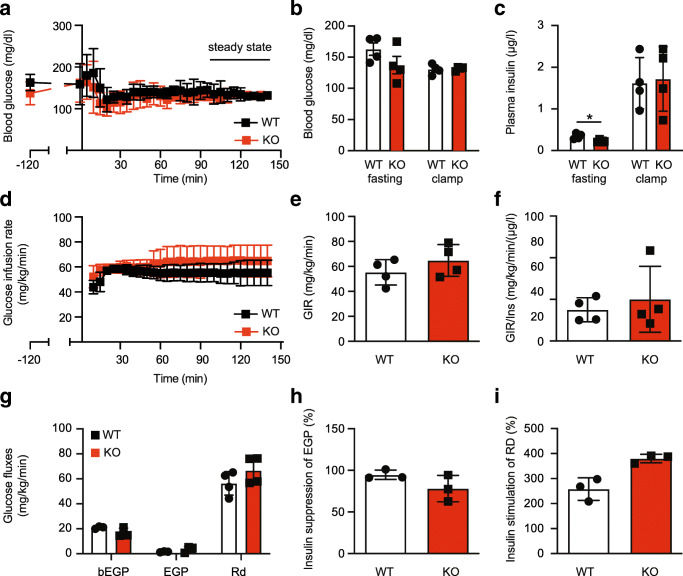
No alteration of tissue-specific insulin sensitivity in TMBIM6^−/−^ mice. **a** Blood glucose levels during the hyperinsulinemic-euglycemic clamps. Steady-state conditions were defined as the last 30 min of the clamp. **b** Blood glucose levels during basal (6 h fasted) conditions and during the steady state of the clamp. **c** Plasma insulin levels during basal conditions and at the end of the clamp. **d** Glucose infusion rates required to maintain euglycemia during hyperinsulinemic clamp (GIR_HE_). **e** Average GIR_HE_ during the steady state of the hyperinsulinemic-euglycemic clamps. **f** GIR_HE_ normalized to the plasma insulin levels at the end of the clamp. **g** Glucose fluxes calculated from D-[6,6-^2^H_2_] glucose dilution during basal (basal endogenous glucose production, bEGP) and insulin-stimulated (endogenous glucose production, EGP; glucose turnover, Rd) conditions. **h** Percent suppression of EGP by insulin compared to basal conditions. **i** Percent stimulation of Rd by insulin compared to basal conditions. Data are presented as mean ± SD and individual data points. Statistical testing using the Student’s *t* test revealed no significant differences

To clarify whether changes in insulin sensitivity are relevant for these differences, we first assessed tissue-specific insulin sensitivity in vivo using hyperinsulinemic-euglycemic clamps. Blood glucose levels were clamped at ~ 125 mg/dL (Fig. 3a). Basal (6 h fasted) as well as clamp (steady state) blood glucose (Fig. 3b) levels were similar between TMBIM6^−/−^ and WT mice while basal plasma insulin but not clamp insulin levels were lower in TMBIM6^−/−^ mice (Fig. 3c). Glucose infusion rate during hyperinsulinemia-euglycemia, which reflects whole-body insulin sensitivity, was comparable between genotypes during the whole clamp (Fig. 3d), steady state (Fig. 3e), and when normalized to plasma insulin levels at the end of the clamp (Fig. 3f). Basal endogenous glucose production (bEGP) and endogenous glucose production (EGP) and glucose turnover (Rd) during hyperinsulinemia were not significantly different (Fig. 3g). Also, the insulin-mediated suppression of EGP (Fig. 3h), reflecting hepatic insulin sensitivity, and insulin-stimulated Rd, mainly reflecting skeletal muscle insulin sensitivity, were comparable between TMBIM6^−/−^ and WT mice (Fig. 3i). We therefore turned to differences in glucose metabolism and quantified glucose-stimulated insulin secretion in vivo using hyperglycemic clamps.

### TMBIM6^−/−^ mice show greater glucose-stimulated insulin secretion in vivo

**Fig. 4:**
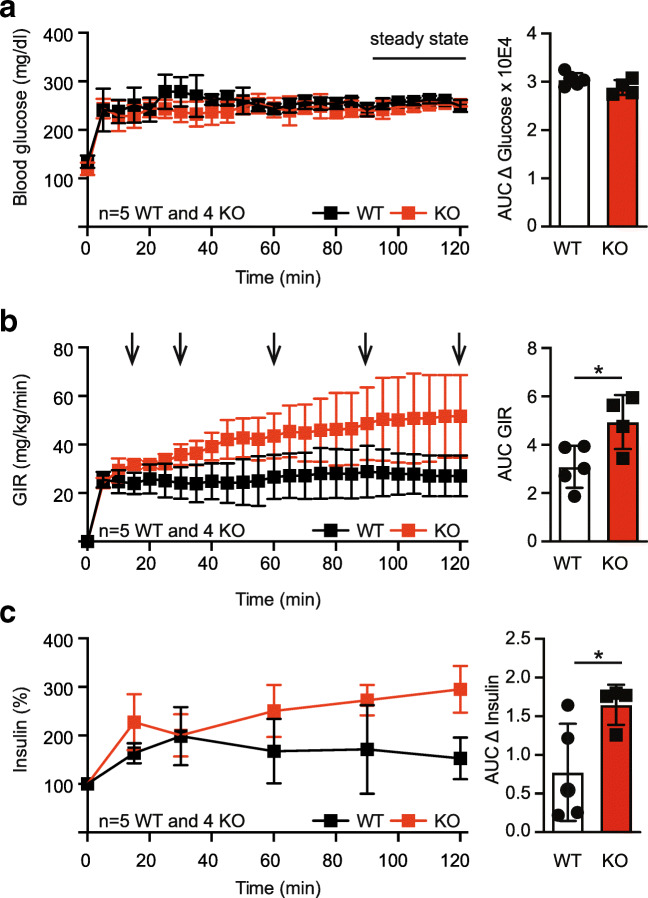
TMBIM6^−/−^ mice show greater glucose-stimulated insulin secretion in vivo. **a** Blood glucose levels during the hyperglycemic clamps. Steady-state conditions were defined as the last 30 min of the clamp. **b** Glucose infusion rates required to maintain hyperglycemia (GIR_HG_). The arrows indicate the time points of blood drawing for measurement of insulin levels during the clamp. **c** Percent change from baseline in insulin concentration during the hyperglycemic clamp. Data are presented as mean ± SD and individual data points. Normal distribution was assessed with the Shapiro-Wilk test and significant differences calculated using the Student’s *t* test in **b** and the Mann-Whitney test in **c**, *p < 0.05

In these experiments, plasma glucose is acutely raised to 125 mg/dL above basal levels by a continuous infusion of glucose. The target blood glucose levels of ~ 250 mg/dL were achieved within the first 60 min and remained stable to the end of the experiment. Importantly, the blood glucose concentrations during the steady state of the clamp (90–120 min) were comparable between WT and TMBIM6^−/−^ mice (Fig. 4a). Glucose infusion rate required to maintain hyperglycemia (GIRHG) during the steady state was ~ 87% higher in TMBIM6^−/−^ compared to WT mice (Fig. 4b) suggesting increased glucose-stimulated insulin secretion and/or better insulin sensitivity in TMBIM6^−/−^. The percent increase of insulin from baseline was higher in TMBIM6^−/−^ mice at 90 min and 120 min of the hyperglycemic clamp (Fig. 4c). Moreover, the area under the plasma insulin curve (AUC) over 120 min was by ~ 112% higher in TMBIM6^−/−^ mice (Fig. 4c) indicating higher glucose-stimulated insulin secretion in these mice.

### Altered glucose-induced Ca^2+^ oscillations in pancreatic islets of TMBIM6^−/−^ mice

**Fig. 5:**
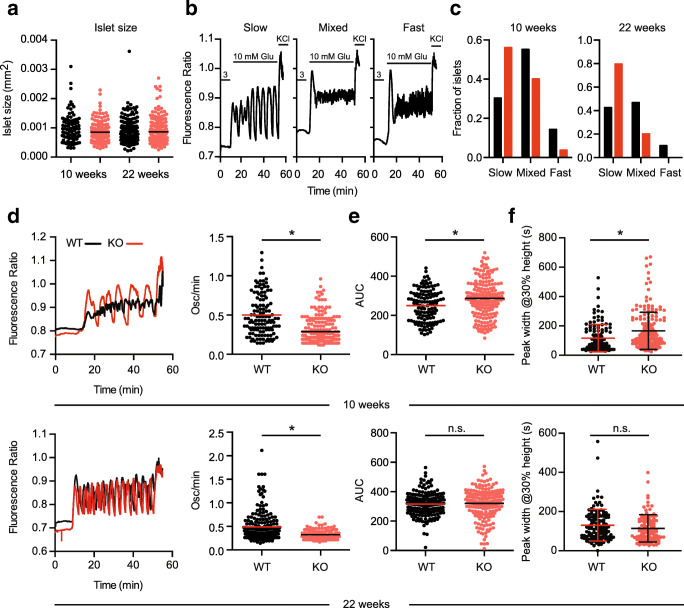
Altered glucose-induced Ca^2+^ oscillations in pancreatic islets of TMBIM6^−/−^ mice. **a** Similar islet size in young and adult KO and WT mice. **b** Typical slow, mixed, and fast Ca^2+^ oscillations observed when perfusing pancreatic islets with low (3 mM), high (10 mM) glucose containing solution and with a 30 mM KCl containing solution as indicated. **c** Dividing the islets from WT and KO mice into slow, fast, and mixed oscillating islets based on the relevant peaks in the Fourier transformed frequency spectrum between 0 and 0.015 Hz (slow) and 0.015 Hz and 0.05 Hz (fast). **d** Superimposed typical example traces of glucose-induced islet Ca^2+^ oscillations from WT and KO mice and oscillation frequency represented as oscillations per minute for each islet and mean. **e** Individual values and mean of the integrated area under the curve of the baseline (100–500 s)-corrected fluorescence ratio trace. **f** Width of Ca^2+^ oscillations at 30% of the peak height for the islets of TMBIM6^−/−^ and WT mice of 10 weeks and **d** 22 weeks. Normal distribution was assessed with the Shapiro-Wilk test and significant differences calculated using the Student’s *t* test, *p < 0.05

To identify the source of the hyperinsulinemia in TMBIM6^−/−^ mice, we turned to pancreatic β cells which secrete insulin in response to changes in glucose levels. The insulin secretion is initiated by an elevation of the intracellular free Ca^2+^ concentration caused by a combination of influx through voltage-gated Ca^2+^ channels and ER Ca^2+^ store release [21]. We hypothesized that changes in glucose-induced Ca^2+^ transients underlie the altered insulin secretion in TMBIM6^−/−^ mice and compared the morphology and Ca^2+^ dynamics of pancreatic islets from young (10 weeks of age) and older (22 weeks) WT and KO mice. Islets were isolated and their size measured, revealing no differences between all groups (Fig. 5a). We then analyzed Ca^2+^ dynamics and oscillating patterns by perfusing FURA2-AM-stained islets with a solution containing 3 mM glucose resembling fasting conditions in vivo as baseline condition and changed to 10 mM glucose to induce intracellular Ca^2+^ oscillations in the islets. This was followed by adding a solution containing 30 mM KCl to evoke depolarization and Ca^2+^ influx at the end of the experiment after 50 min. By taking the Fourier transformation of the first-time derivative of the Ca^2+^ trace and normalizing this for the frequencies between 0 and 0.05 Hz [22], we binned islets showing peaks > 0.6 only in the slow part of the spectrum (0–0.015 Hz), only in the fast part of the spectrum (0.015–0.05 Hz), or in both (mixed) parts of the frequency spectrum [23] (exemplary traces in Fig. 5b). This revealed an increase in islets with slow oscillations and a slower overall oscillation frequency in TMBIM6^−/−^ animals in both age groups (Fig. 5 c and d). The area under the curve, defined as the integration of the baseline-corrected Ca^2+^ trace from 0 to 50 min, is a value for the total intracellular Ca^2+^ during the measurement. This demonstrated a significant higher total value in young TMBIM6^−/−^ mice compared to young WT but no difference in old animals (Fig. 5e). We measured the width of the Ca^2+^ peaks in the oscillation patterns, which confirmed an increase in the average width of the oscillations in the 10-week-old TMBIM6^−/−^ compared to the WT islets (Fig. 5f). There is a longer plateau phase of high intracellular Ca^2+^ in the absence of TMBIM6; this difference is no longer observed in 22-week-old mice. This increased peak width explains why the AUC Ca^2+^ is higher despite the lower frequency of oscillations.

### Increased IRE1α levels but no changes in *Xbp1* splicing or Ire1-dependent decay in TMBIM6^−/−^ mice

**Fig. 6:**
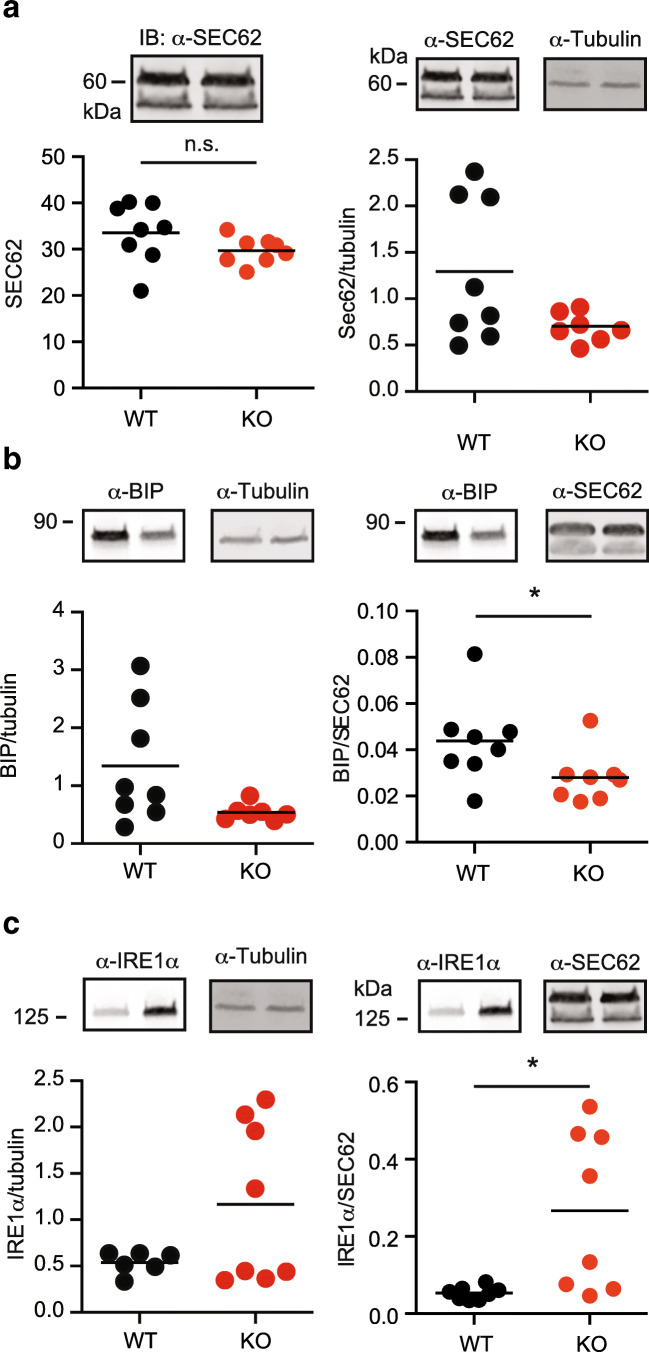
Increased IRE1α levels in TMBIM6^−/−^ mice. **a** Immunoblotting demonstrating no significant regulation of the ER resident protein SEC62 normalized to α/β-tubulin or protein weight. **b** Reduced expression of the important ER chaperone BIP (GRP78) and **c** increased expression of IRE1α in the pancreas from old mice when normalized to SEC62 but not to α/β-tubulin. Size is indicated. Each data point represents data obtained from one mouse. Normal distribution was assessed using the Shapiro-Wilk test and significant differences calculated using the non-parametric Mann-Whitney test, *p < 0.05

**Fig. 7:**
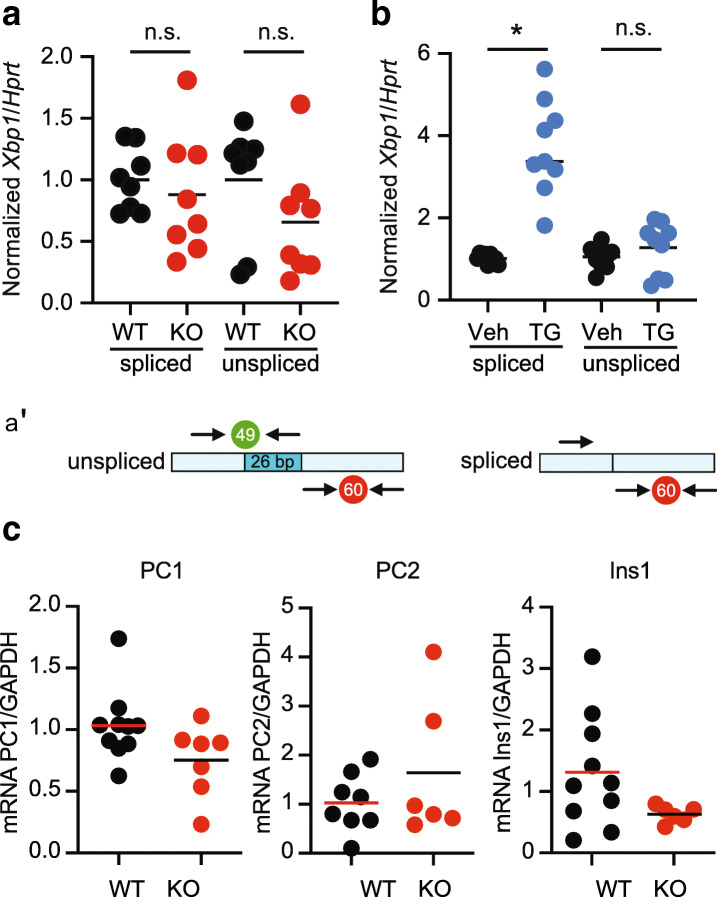
No alterations in *Xbp1* splicing or Ire1-dependent decay in TMBIM6^−/−^ mice. **a** Unaltered amounts of spliced and total *Xbp1* shown by quantitative RT-PCR using probes specific for unspliced or total (spliced and unspliced) *Xbp1*. **a′** Scheme illustrates the position of primers and probes in regard to the spliced part of *Xbp1*. **b** Mouse embryonic fibroblasts treated with 0.5 μM thapsigargin served as positive control for the assay. **c** Quantitative RT-PCR demonstrating similar mRNA levels of the indicated genes. Each data point represents data obtained from one mouse. Normal distribution was assessed using the Shapiro-Wilk test and significant differences calculated using the non-parametric Mann-Whitney test, *p < 0.05

To investigate the relevance of ER stress to the observed phenotype, we quantitated the expression levels of the ER luminal chaperone BiP and the ER stress sensor IRE1α in pancreatic tissue of old mice (> 22 weeks) using immunoblotting. BiP binds the ER lumenal domains of all three stress sensors, IRE1α, PERK, and ATF6, in the unstressed state and is induced by all three sensors although to a different degree [1]. We further focused on IRE1α, as it was previously shown to be overactive in TMBIM6^−/−^ animals under conditions of low level ER stress [10] and because TMBIM6 was previously shown to directly bind and suppress IRE1α activity [10]. All blots were normalized to the housekeeping protein α/β-tubulin and the ER resident protein Sec62 which served to rule out differences caused by ER expansion. Sec62 is a suitable control for this as it was recently shown not to be induced by ER stress [24]. Sec62 itself was normalized to protein weight and α/β-tubulin and had similar expression levels in TMBIM6^−/−^ mice as compared to wild type (Fig. 6a). Interestingly, when normalized to Sec62, our experiments revealed a statistically significant downregulation of BIP in TMBIM6^−/−^ mice (Fig. 6b); thus, the opposite result one would expect in the case of a constitutive activation of ER stress and an upregulation of IRE1α (Fig. 6c). When compared to α/β-tubulin, neither BiP nor IRE1α were, however, significantly differently expressed (Fig. 6 b and c) although of course the same tendency prevailed. This upregulation of IRE1α did, however, not result in increased splicing of the target of its endoribonuclease activity, *Xbp1* in pancreas tissue (Fig. 7a). In these experiments, we used two independent primer and probe-based real-time PCR assays, one that detects total (thus spliced and unspliced) *Xbp1* mRNA and one that detects only unspliced *Xbp1* because the reverse primer for this assay binds on the spliced-out fragment of 26 bp (see scheme in Fig. 7a′). This assay is capable of detecting *Xbp1* mRNA splicing under conditions of ER stress elicited by the sarco/endoplasmic reticulum ATPase (SERCA) inhibitor thapsigargin (Fig. 7b) proving the feasibility of this approach. Finally, as IRE1α activation can also induce the degradation of specific mRNAs through a pathway called regulated Ire1-dependent decay (RIDD) [3, 4], we decided to quantitate the expression levels of such RIDD-target mRNAs including proprotein convertase 1 and 2 (*PC1* and *PC2*) and insulin (*Ins1)*, which are involved in the insulin secretory pathway [25]. This revealed no significant changes in the abundance of these mRNAs in TMBIM6^−/−^ mice (Fig. 7c).

Based on these observations, we conclude that ER stress is not induced constitutively—thus without additional stressor—in the pancreas of TMBIM6^−/−^ mice and probably less relevant for the observed phenotype.

## Discussion

This study demonstrates that TMBIM6^−/−^ mice develop obesity and hepatic steatosis not by a decreased energy expenditure or altered muscle and liver insulin resistance but rather by increased insulin-dependent fat storage caused by changes in glucose-mediated Ca^2+^ oscillations in pancreatic β cells.

TMBIM6 has been implicated with obesity and diabetes before, as it is downregulated in liver and muscle of genetically obese ob/ob and db/db mice as well as in mice with diet-induced obesity [17]. On the other hand, overexpression of TMBIM6 by adenoviral gene transfer reversed hyperglycemia in normal mice and in diet-induced obese mice which was partially explained by an increased insulin sensitivity and reduction of hepatic glucose production [17]. We show that TMBIM6 deficiency alters glucose-dependent Ca^2+^ signaling in pancreatic β cells thereby instigating increased insulin secretion, a well-established direct cause for obesity and hepatic steatosis [26].

Pancreatic β cells are among the most differentiated and regulated cells in the body. Insulin secretion is tightly controlled by glucose sensing and by hormonal and neuronal regulation. Changes in intracellular Ca^2+^ are the immediate trigger for secretory events in the β cells. The main changes in cytoplasmic Ca^2+^ concentrations occur due to the interplay between extracellular Ca^2+^ influx through voltage-gated Ca^2+^ channels and efflux through the Na^+^/Ca^2+^ exchanger NCX1. Another important pathway that increases [Ca^2+^]_cyt_ is Ca^2+^ release from intracellular stores. In the ER, Ca^2+^ is highly buffered with Ca^2+^ binding proteins, and relative small changes in the [Ca^2+^]_ER_ can profoundly affect [Ca^2+^]_cyt_. The ER is a very leaky organelle in terms of Ca^2+^ release, which is illustrated by the rapid depletion of ER Ca^2+^ upon inhibiting reuptake with thapsigargin [27]. During [Ca^2+^]_cyt_ oscillations, the [Ca^2+^]_ER_ oscillates in phase effectively buffering the [Ca^2+^]_cyt_. At the end of a [Ca^2+^]_cyt_ transient, a slow Ca^2+^ release from the ER contributes to longer oscillations [28]. We observed that after glucose stimulation, intracellular Ca^2+^ in TMBIM6^−/−^ islets oscillates with a lower frequency than WT islets, but Ca^2+^ peaks are wider. This leads to an increased area under the curve of the glucose-induced Ca^2+^ signal in pancreatic islets from TMBIM6^−/−^. As such periods of higher intracellular Ca^2+^ coincide with higher insulin secretion [29], we conclude that TMBIM6^−/−^ islets secrete more insulin which is in line with the hyperinsulinemia observed during our hyperglycemic clamp experiments.

The increased [Ca^2+^]_cyt_ in the β cells is in striking similarity to the phenotype observed in TMBIM6^−/−^ splenocytes [16]. In accordance with these results, it is tempting to speculate that under basal conditions, TMBIM6 participates in the ER Ca^2+^ leak and mediates Ca^2+^ release from the ER, effectively reducing the intraluminal Ca^2+^. Subsequently, under stimulatory conditions, there is less Ca^2+^ release in WT compared to TMBIM6^−/−^ cells [30]. The reduced leak of Ca^2+^ from the ER results in more Ca^2+^ available for glucose-induced Ca^2+^ release which correlates with an increased insulin secretion in the TMBIM6^−/−^ mice. Store-operated Ca^2+^ release in the glucose-stimulated β cell is highly dependent on ER Ca^2+^ filling and small variations in [Ca^2+^]_ER_ can therefore result in larger Ca^2+^ transients and increased insulin secretion [31].

The role of TMBIM6 has been predominantly studied in relation to ER stress and the unfolded protein response [10]. Our observations are in accordance with increased [Ca^2+^]_ER_ in TMBIM6^−/−^ islets which appears independent of IRE1α-mediated ER stress. We found no evidence of continuous ER stress in pancreatic tissue; the major ER chaperone BIP was downregulated, and despite an increased abundance of IRE1α, no changes in *Xbp1* splicing or increased RIDD were detected. It is possible that the direct interaction and inhibition of IRE1α by TMBIM6 reported previously [10] cause an adaptive upregulation of IRE1α which does not seem to translate into an increased endoribonuclease or RIDD activity of IRE1α but represents a new homeostatic equilibrium. It is conceivable that the upregulation of IRE1α is due to its recently described non-canonical function in mitochondria-associated endoplasmic reticulum composition which seems to play a role in the control of Ca^2+^ transfer and bioenergetics [32] which would fit our observations of predominant changes in Ca^2+^ handling in TMBIM6^−/−^ mice under steady-state conditions. Overall, our results are well in line with previously reported effects of TMBIM6 in the control of the ER Ca^2+^ content in vitro [7, 11, 12, 14] and in vivo [16] where TMBIM6 was found to affect lymphocyte function by perturbing cytosolic and ER Ca^2+^ levels without affecting the ER stress response. In splenocytes, we even found a downregulation of the UPR target protein ERp57 [16] similar to the downregulation of BiP observed here. Also, structural evidence obtained from a bacterial homolog of TMBIM6 suggests that TMBIM6 is mainly a pH-sensitive Ca^2+^ leak channel [15].

Interestingly, TMBIM6^−/−^ mice developed obesity and hepatic steatosis without evidence for hepatic or whole-body insulin resistance. Whole-body energy metabolism as well as caloric intake was not altered precluding changes in energy balance as the cause of the increased ectopic fat storage. Such dissociation between insulin resistance and steatosis has been also reported for other gene variants such as PNPLA3 [33]. We suggest that hyperinsulinemia stimulates triglyceride synthesis and thereby induces weight gain [34]. Of note, chronic hyperinsulinemia in insulinoma also frequently associates with weight gain [35]. In addition, altered calcium fluxes modulating hepatic ER stress, mitochondrial function, or inflammatory pathways could also contribute to hepatic steatosis and even increase the risk for its progression [36–38].

These findings highlight the importance of TMBIM6 in the development of obesity and hepatic steatosis. Our study suggests that loss-of-function of TMBIM6 might contribute to the development of metabolic syndrome and further understanding of its role will contribute to the development of novel strategies to prevent or treat this serious condition.
